# Growth promotion of the opportunistic human pathogen, *Staphylococcus lugdunensis*, by heme, hemoglobin, and coculture with *Staphylococcus aureus*

**DOI:** 10.1002/mbo3.162

**Published:** 2014-02-07

**Authors:** Jeremy R Brozyna, Jessica R Sheldon, David E Heinrichs

**Affiliations:** 1 Department of Microbiology and Immunology, University of Western OntarioLondon, Ontario, Canada, N6A 5C1; 2 Centre for Human Immunology, University of Western OntarioLondon, Ontario, Canada, N6A 5C1

**Keywords:** Heme, hemoglobin, iron acquisition, staphylococci, staphyloferrins.

## Abstract

*Staphylococcus lugdunensis* is both a commensal of humans and an opportunistic pathogen. Little is currently known about the molecular mechanisms underpinning the virulence of this bacterium. Here, we demonstrate that in contrast to *S. aureus*,*S. lugdunensis* makes neither staphyloferrin A (SA) nor staphyloferrin B (SB) in response to iron deprivation, owing to the absence of the SB gene cluster, and a large deletion in the SA biosynthetic gene cluster. As a result, the species grows poorly in serum-containing media, and this defect was complemented by introduction of the *S. aureus*SA gene cluster into *S. lugdunensis*. *S. lugdunensis* expresses the HtsABC and SirABC transporters for SA and SB, respectively; the latter gene set is found within the *isd* (heme acquisition) gene cluster. An *isd* deletion strain was significantly debilitated for iron acquisition from both heme and hemoglobin, and was also incapable of utilizing ferric-SB as an iron source, while an *hts* mutant could not grow on ferric-SA as an iron source. In iron-restricted coculture experiments, *S. aureus* significantly enhanced the growth of *S. lugdunensis*, in a manner dependent on staphyloferrin production by *S. aureus*, and the expression of the cognate transporters by *S. lugdunensis*.

## Introduction

Twenty-five years ago, *Staphylococcus lugdunensis* was described as a new species of coagulase-negative staphylococcus (CoNS), isolated from a human clinical specimen (Freney et al. 1988). It is now widely considered to be an emerging pathogen with uncharacteristically elevated virulence in comparison with other members of the CoNS (Rosenstein and Götz 2013). In addition to being a skin commensal, *S. lugdunensis* is responsible for both nosocomial and community-acquired infections that may include skin and soft tissue infections (SSTIs), pneumonia, meningitis, and endocarditis (Sotutu et al. 2002; Anguera et al. 2005; Arias et al. 2010; Kleiner et al. 2010). While the most common clinical manifestation of *S. lugdunensis* infection is SSTIs (55.4%), blood infections and those associated with vascular catheterization accounted for a notable 17.4% of diagnoses (Herchline and Ayers 1991). Strikingly, the mortality rate of *S. lugdunensis-*associated endocarditis may reach up to 50% (Anguera et al. 2005). Despite that *S. lugdunensis* is gaining notoriety as an atypically virulent CoNS, the true burden of *S. lugdunensis* infection is likely underestimated. Most *S. lugdunensis* isolates are hemolytic, and although do not secrete soluble coagulase, do produce a membrane-bound clumping factor (coagulant), therefore, it is possible that many *S. lugdunensis* infections are misinterpreted as being caused by *S. aureus* (Frank et al. 2008; Böcher et al. 2009; Szabados et al. 2011). Moreover, nearly half of patients infected with *S. lugdunensis* appear to have no comorbidities, indicating that this underappreciated pathogen is able to cause infection in the absence of overt susceptibility (Kleiner et al. 2010).

Iron is an essential nutrient for most pathogenic bacteria, including the Staphylococci, and represents a significant growth-limiting nutrient in the host (Ratledge and Dover 2000). Virtually all host iron is bound to glycoproteins such as transferrin, ferritin, and lactoferrin (Gomme et al. 2005), or is in complex with heme in hemoproteins. Hemoglobin iron accounts for up to 75% of total host iron, the vast majority of which is found within circulating erythrocytes (Pishchany and Skaar 2012). To establish infection, pathogens must circumvent host iron sequestration strategies, and therefore, by extension, must possess elaborate iron acquisition mechanisms in order to obtain this limited nutrient. Frequently, these iron uptake strategies involve either the acquisition of heme contained in hemoglobin, or the removal of transferrin-bound iron through the secretion of siderophores (Hood and Skaar 2012). Siderophores are small molecules (commonly less than 1000 Da) capable of binding ferric iron with high affinity, and delivering iron back to the cell via surface localized and membrane-embedded receptor proteins.

Much of our molecular understanding of iron acquisition processes in the staphylococci comes from studies in *S. aureus*. The iron-regulated surface determinants (Isds) were first discovered in *S. aureus* (Mazmanian et al. 2002; Mazmanian et al. 2003). The Isd system is now fairly well-characterized, and is constituted by a series of proteins that, together, are capable of extracting heme from hemoglobin at the bacterial cell surface, and relaying heme across the cell wall, through the cytoplasmic membrane, and into the cytoplasm where it is degraded to release iron for use in cellular processes (Skaar and Schneewind 2004; Grigg et al. 2010ac2010c). *S. aureus* also produces two siderophores, staphyloferrin A (SA) and staphyloferrin B (SB), which are synthesized by gene products encoded from within the *sfa* and *sbn* genetic loci, respectively (Beasley and Heinrichs 2010; Hammer and Skaar 2011). *S. aureus* internalizes ferric-SA and ferric-SB using the ABC-type transporters HtsABC and SirABC, respectively, encoded by genes found adjacent to the cognate biosynthetic loci (Beasley and Heinrichs 2010; Grigg et al. 2010a,b; Beasley et al. 2011a). *S. aureus* strains mutated for staphyloferrin production are severely restricted for growth in the presence of transferrin or animal serum (Beasley et al. 2009, 2011a,b).

Investigations of the molecular mechanisms that contribute to the virulence of *S. lugdunensis* are in their infancy. Few mutants have, as of yet, been constructed and characterized, and even fewer tested in animal models. In one recent study, it was demonstrated that in *S. lugdunensis*, sortase A, responsible for the anchoring of LPXTG-containing proteins to the cell wall, was required for full virulence in a rat endocarditis model (Heilbronner et al. 2013). Interestingly, the genome sequencing of two strains of *S. lugdunensis*, HKU09-01 and N920143, revealed that this species is unique among the CoNS in that it encodes an Isd system (Tse et al. 2010; Haley et al. 2011; Heilbronner et al. 2011; Zapotoczna et al. 2012) and, moreover, in strain HKU09-01, the *isd* locus is tandemly duplicated. Work led by T. Foster's group has revealed that the *isd* system in strain N920143 is functional, contributing to the strain's use of hemoglobin as an iron source (Zapotoczna et al. 2012).

In this study, we investigate iron uptake mechanisms in *S. lugdunensis* through characterizing the role of the *S. lugdunensis* strain HKU09-01 *isd* locus in heme and hemoglobin utilization, as well as characterizing this species for the ability to produce and utilize staphyloferrins. We demonstrate that a mutant lacking *isd* is severely impaired for growth using heme and hemoglobin as a sole iron source, especially at nanomolar heme and hemoglobin concentrations. Moreover, we show that *S. lugdunensis* grows poorly in serum and in the presence of transferrin owing to a lack of detectable siderophore production. We further demonstrate that while *S. lugdunensis* cannot produce staphyloferrins, it encodes the transporters for their uptake and these transporters are functional, leading to the notion that *S. lugdunensis* may appropriate staphyloferrins from other staphylococcal species to augment its growth. In support of this, we show that growth of iron-restricted *S. lugdunensis* is significantly enhanced in coculture with staphyloferrin-producing *S. aureus*, in an *hts-* and *sir*-dependent manner.

## Experimental Procedures

### Bacterial strains and growth conditions

Bacterial strains and plasmids used in this study are summarized in Table 1. For all routine manipulations, *Escherichia coli* DH5*α* was grown in Difco Luria–Bertani broth (LB; BD-Canada, Mississauga, ON, Canada) or on LB agar (LBA). *S. lugdunensis* and *S. aureus* strains were cultured in tryptic soy broth (TSB; BD Diagnostics) or on TSB agar (TSA). Antibiotics were used at the following concentrations: 100 *μ*g/mL ampicillin for *E. coli* selection; 10 *μ*g/mL chloramphenicol for *S. lugdunensis* selection. For subsequent experiments, *S. lugdunensis* and *S. aureus* were grown in several different iron-restricted media as detailed: (i) Tris-minimal succinate broth (TMS) (Sebulsky et al. 2004); (ii) TMS treated with Chelex-100 resin (Bio-Rad, Mississauga, ON, Canada) for 24 h at 4°C (C-TMS); (iii) an 80:20 mixture of C-TMS to complement-inactivated horse serum (Sigma Aldrich, Oakville, ON, Canada); or (iv) RPMI media 1640 (Life Technologies, Burlington, ON, Canada) reconstituted from powder and supplemented with 1% w/v casamino acids (RPMIC) and 1 *μ*mol/L of the iron chelator ethylenediamine-di(*o*-hydroxyphenylacetic acid) (EDDHA; LGC Standards GmbH, Teddington, Middlesex, U.K.). All bacteria were cultured at 37°C, shaking at 200 rpm, unless otherwise indicated. All media and solutions were prepared with water purified through a Milli-Q water purification system (EMD Millipore, Billerica, MA).

**Table 1:** Bacterial strains, plasmids, and oligonucleotides used in this study.

Bacterial strain, plasmid or oligonucleotide	Description^1^	Source or reference
Strains		
*E. coli*		
DH5*α*	*φ*f80dlacZΔM15 recA1 endA1 gyrAB thi-1 hsdR17( 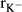 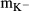 ) supE44 relA1 deoR *Δ(lacZYA-argF)U169*	Promega
*Staphylococcus lugdunensis*		
HKU09-01	Human skin infection isolate	Tse et al. (2010)
H2710	HKU09-01 Δ*isd*-*sir*	This study
H2773	HKU09-01 Δ*htsABC*	This study
H2774	HKU09-01 Δ*isd*-*sir* Δ*htsABC*	This study
*Staphylococcus aureus*		
RN4220	Prophage-cured laboratory strain; 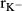 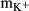 ; accepts foreign DNA	Kreiswirth et al. (1983)
RN6390	Prophage-cured laboratory strain	Peng et al. (1988)
H1324	RN6390 Δ*sbn*::Tet; Tet^R^	Beasley et al. (2009)
H1661	RN6390 Δ*sfa*::Km; Km^R^	Beasley et al. (2009)
H1649	RN6390 Δ*sbn*::Tet Δ*sfa*::Km; Tet^R^ Km^R^	Beasley et al. (2009)
H306	RN6390 Δ*sirA*::Km; Km^R^	Beasley et al. (2009)
H1448	RN6390 Δ*hts*::Tet; Tet^R^	Beasley et al. (2009)
Plasmids		
pKOR1	*E. coli/Staphylococcus* shuttle vector allowing allelic replacement in staphylococci	Bae and Schneewind (2006)
pKOR1Δ*isd-sir*	pKOR1 plasmid for deletion of duplicated genetic region encompassing *isd* and *sir*	This study
pKOR1Δ*hts*	pKOR1 plasmid for in-frame deletion of *htsABC*	This study
pRMC2	*E. coli/Staphylococcus* shuttle vector: Ap^R^ Cm^R^	Corrigan and Foster (2009)
pRMC2::*sir*	pRMC2 derivative for *sirABC* expression; Cm^R^	This study
pRMC2::*hts*	pRMC2 derivative for *htsABC* expression; Cm^R^	This study
pLI50	*E. coli/Staphylococcus* shuttle vector: Ap^R^ Cm^R^	Lee and Iandolo (1986)
pLI50::*sfaABCD* (pEV90)	pLI50 derivative containing *sfaABCD* from *S. aureus*; Cm^R^	Beasley et al. (2009)

Oligonucleotides^2^,^3^ purpose	Sequence (5′–3′)
Primers for generating upstream and downstream recombinant regions for Δ*isd* Δ*sir* using pKOR1	(*AttB1*)-isdUF:*GGGGACAAGTTTGTACAAAAAAGCAGGCT* CTACCACTGACAGCAACGGCAAT
	*isdUR: CTCATTGCGATTCCTTCCTTCG*
	*isdDF: Phos/AGCACAGATAGGAGTTCATTTGCATGTA*
	*isdDF: Phos/AGCACAGATAGGAGTTCATTTGCATGTA*
	(AttB2)-isdDR:*GGGGACCACTTTGTACAAGAAAGCTGGGT* AGATGCGCCTTGGATTTGACAC
Primers for generating upstream and downstream recombinant regions for Δ*hts* using pKOR1	(*AttB1*)-htsUF:*GGGGACAAGTTTGTACAAAAAAGCAGGCT* ATTCCAGTATGTTGCCAC
	*htsUR: AACAGTAGCCCAATGATAC*
	*htsDF: Phos/AGTAGGTGTCATAATAGC*
	(*AttB2*)-htsDR:*GGGGACCACTTTGTACAAGAAAGCTGGGT* TACTGGTAATGAGCACTC
Primers for cloning *sirABC* into pRMC2 for complementation	XhoI-sirF: GATCCTCGAGTACTGCTCCAAAATCCCC
	EcoRI-sirR: GATCGAATTCTTTAGCGTGCGGTATGTC
Primers for cloning *htsABC* into pRMC2 for complementation	KpnI-htsF: GATCGGTACCAAGCACTAACCCAGTCAATG
	SacI-htsR: GATCGAGCTCCTAAACAATCCCCATAAAGC

1 Ap^R^, Cm^R^, Km^R^, and Tet^R^: resistance to ampicillin, chloramphenicol, kanamycin, and tetracycline, respectively.

2 Restriction sites for cloning are underlined.

3 Phos/denotes a 5′ phosphate on the primer.

### Generation of Δ*isd-sir* and *ΔhtsABC* mutants in *S. lugdunensis*

**Figure 1:**
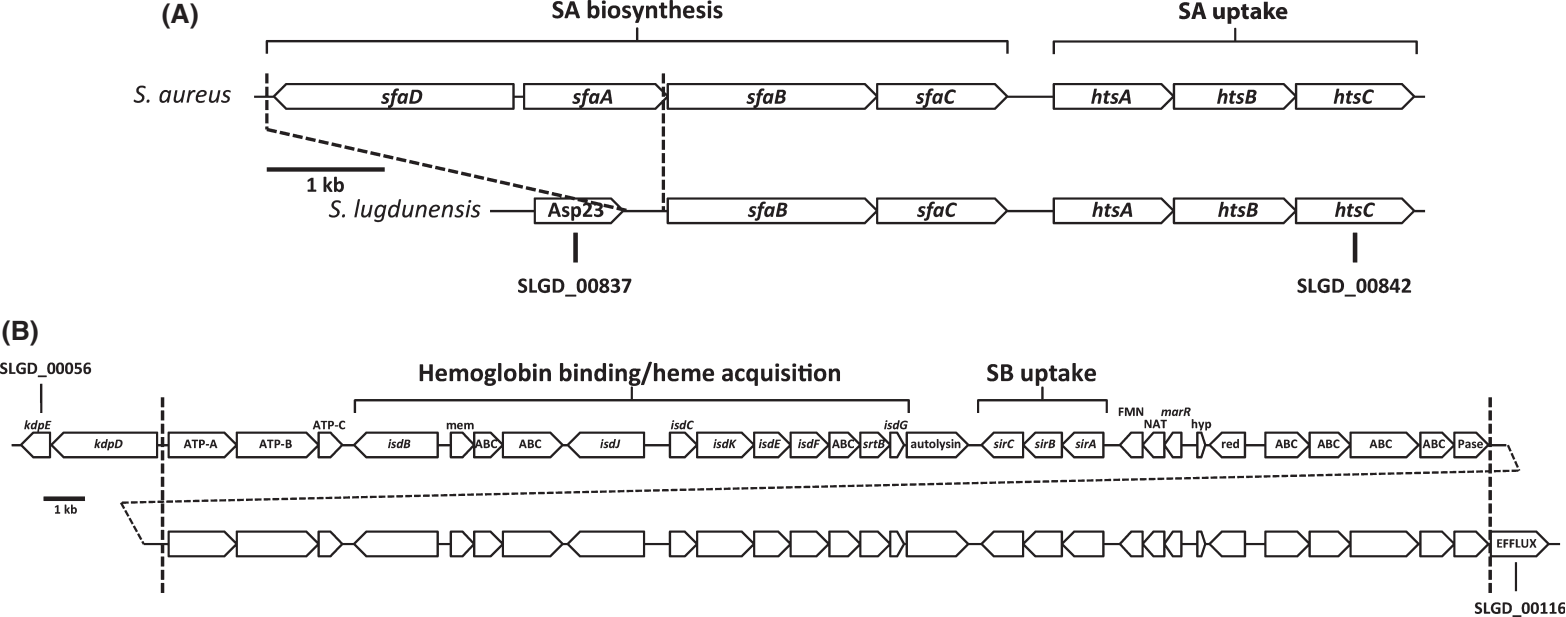
Physical maps of the *sfa-hts* and *isd* loci in *Staphylococcus lugdunensis*. (A) Staphyloferrin A (SA) biosynthetic and uptake locus. Shown is the homologous locus in *S. aureus* versus that which is present in all sequenced genomes of *S. lugdunensis*. Note that the SA biosynthetic locus in *S. lugdunensis* carries a deletion that eliminates two genes completely (*sfaA* and *sfaD*), along with the promoter for the remaining two genes (*sfaB* and *sfaC*). The deleted region is indicated between the dashed lines. Asp23, alkaline shock protein 23. (B) Shown is the ∼65-kb region of the *S. lugdunensis* strain HKU09-01 genome (spanning orfs SLGD_00056 to SLGD_00116) with the tandemly duplicated *isd-sir* locus. The duplicated region is shown between the dashed vertical lines. Abbreviations are as follows, with predicted or hypothetical functions: ABC, component of an ATP-binding cassette transporter; ATP-A,B,C, K+-ATPase components A, B, and C, respectively; mem, membrane protein; FMN, FMN binding protein; NAT,*N*-acetyltransferase; marR, MarR-type regulator; hyp, hypothetical; Red, reductase; Pase, phosphatase.

Primer sequences used for the construction and complementation of *S. lugdunensis* mutants can be found in Table 1. Allelic replacement was performed as previously described, using the plasmid pKOR1 (Bae and Schneewind 2006). In brief, 500–1000-bp DNA fragments were amplified from the genomic regions upstream and downstream of the tandem-duplicated *isd-sir* locus (Fig. 1B) using the primers isdUF and isdUR, and isdDF and isdDR, respectively. The amplicons were cloned into pKOR1, generating pKOR1Δ*isd*-*sir*. Similarly, the plasmid pKOR1Δ*hts* for the in-frame deletion of *htsABC* (Fig. 1A) was constructed by amplifying 500–1000-bp DNA fragments flanking the start and stop codons of the operon using primers htsUF and htsUR, and htsDF and htsDR. The vectors were passaged through *S. aureus* RN4220 (a restriction-negative, modification-positive strain) before introduction into *S. lugdunensis* HKU09-01 by electroporation (Monk et al. 2012). The strains HKU09-01 Δ*isd*-*sir* (H2710) and HKU09-01 Δ*htsABC* (H2773) were generated using the methodology for pKOR1 (Bae and Schneewind 2006; Zapotoczna et al. 2012), and introduction and recombination of pKOR1Δ*hts* with H2710 was used to produce the *isd, sir, hts*-deficient strain H2774. Chromosomal deletions were confirmed through sequencing of PCR amplicons generated from across the deleted regions.

### Complementation of *sirABC* and *htsABC* mutations

For complementation of the *S. lugdunensis sirABC* deletion, primers XhoI-SirF and EcoRI-SirR were used to amplify the wild-type *sirABC* operon, including its native promoter. The fragment was cloned into pRMC2 generating pRMC2::*sir*. The *htsABC* complementation vector pRMC2::*hts* was similarly created using the amplicon generated with oligonucleotides KpnI-HtsF and SacI-HtsR, again including the native promoter for *htsABC*.

### Bacterial growth curves

Single, isolated *S. aureus* and *S. lugdunensis* colonies, taken from TSA plates after overnight incubation, were resuspended in 120 *μ*L C-TMS and 100 *μ*L of this suspension was used to inoculate 2 mL C-TMS. These cultures were then incubated at 37°C for at least 4 h until OD_600_ was ∼1. The cultures were subsequently normalized to an OD_600_ of 1 and 1 *μ*L was added to 200 *μ*L aliquots of 80:20 C-TMS:horse serum growth media. For iron-replete conditions, 100 *μ*M FeCl_3_ was included. Chloramphenicol was added for strains harboring pLI50, pRMC2 or their derivatives. Cultures were grown with constant shaking at medium amplitude in a Bioscreen C machine (Growth Curves USA, Piscataway, NJ) at 37°C. OD_600_ was measured every 15 min, however, for graphical clarity, measurements at 4-h intervals are shown.

### Siderophore preparations and plate bioassays

SA and SB were synthesized enzymatically as previously described (Cheung et al. 2009; Grigg et al. 2010b). Alternatively, concentrated culture supernatants enriched for SA and SB were prepared from *S. aureus* Δ*sbn* and Δ*sfa* mutants, respectively, as previously described (Beasley et al. 2009). Concentrated culture supernatants from *S. lugdunensis* were similarly prepared. In brief, strains were grown in C-TMS with aeration, for 40 h. Bacterial cells were removed by centrifugation and supernatants were lyophilized overnight and resuspended in methanol (one-fifth the original culture volume). Insoluble material was removed by centrifugation and the soluble fraction was rotary evaporated. Dried material was resuspended in water (one-tenth the original culture volume). The ability of concentrated culture supernatants to support staphylococcal growth was assessed using the plate bioassay technique as previously described (Sebulsky et al. 2000). To assess growth promotion on *S. lugdunensis* cells, 1 × 10^4^ cells/mL were incorporated into TMS-agar containing 5 *μ*mol/L EDDHA. Chloramphenicol was incorporated into media with strains harboring pRMC2, pLI50, or vector derivatives. SA and SB were also synthesized enzymatically, using procedures that have been previously described (Cheung et al. 2009; Grigg et al. 2010b). Siderophores/supernatants were applied to sterile paper disks and placed onto the plates. Growth around disks was measured after 24 h.

### Chrome azurol S assay

Supernatants of iron-starved staphylococci were concentrated by lyophilization to 1/10 of their original volume and tested for iron-binding compounds using the chrome azurol S shuttle solution (Schwyn and Neilands 1987), as previously described (Cheung et al. 2009).

### Analysis of iron-regulated protein expression by Western blotting

Antisera against *S. aureus* HtsA and SirA used in this study were generated previously (Dale 2004; Beasley et al. 2009). The antisera were used to assess the expression of homologous proteins in *S. lugdunensis,* as described below.

For analysis of iron-regulated protein expression in whole-cell lysates, cells were grown in C-TMS with or without 50 *μ*mol/L FeCl_3_ for 24 h, normalized to an OD_600_ of 1, and lysed. Proteins in lysates were resolved through SDS-polyacrylamide gel electrophoresis using a 12% acrylamide resolving gel. For Western immunoblots, proteins were transferred from gels to a 45-*μ*m nitrocellulose membrane via standard protocols (Sambrook et al. 1989). Detection of transferred proteins was performed after blocking the membrane at 4°C for 12 h in phosphate buffered saline (PBS) containing 10% w/v skim milk and 20% v/v horse serum. The membrane was washed and the primary antibody was applied at room temperature for 2 h (1:3000 dilution) in PBS with 0.05% Tween 20, and 5% horse serum. The membrane was washed again, and anti-rabbit IgG conjugated to IRDye 800 (Li-Cor Biosciences, Lincoln, NE) was used as the secondary antibody, applied at room temperature for 1 h (1:20,000 dilution), in the same buffer as the primary antibody. Fluorescence was analyzed on a Li-Cor Odyssey infrared imager (Li-Cor Biosciences).

### Staphylococcal growth in coculture

Staphylococci were grown for 4 h in C-TMS. Cells were washed three times in C-TMS and normalized to an OD_600_ of 1 (*S. lugdunensis*) or 0.1 (*S. aureus*). Staphylococci were inoculated into 2 mL of media (80:20 C-TMS:horse serum) either in monoculture or in coculture. For cocultures, equal volumes of washed cells from each species were added. The 2-mL cultures were in 14-mL round-bottom polypropylene tubes and shaken at 200 rpm. Samples were taken at time 0, 12, and 24 h for dilution and plating on TSA to obtain values for viable colony-forming units (CFUs). TSA plates were incubated at 37°C for 24 h and subsequently at room temperature for 2 days. Staphylococci were distinguished based on colony color and morphology; *S. aureus* colonies were visibly larger in diameter and with lighter pigmentation, whereas *S. lugdunensis* colonies were distinguishably smaller (∼¼–½ the diameter of *S. aureus* colonies) and dark yellow under these culture conditions.

### Preparation of hemin and hemoglobin

A solution of bovine hemin (Sigma Aldrich) was prepared as follows. A stock solution at 5 mmol/L in 0.1 N NaOH is prepared and vigorous vortexing ensures the hemin is solubilized completely. The solution is filtered through 0.2 micron filter. The final concentration of hemin, postfiltration, was determined by making dilutions in 0.1 N NaOH and measuring the UV–Vis spectra, using the molar extinction coefficient for hemin in 0.1 N NaOH of 58,400 cm^−1^ (mol/L)^−1^ at 385 nm. Hemin stocks were stored at −20°C. For use in growth assays, hemin stocks are diluted in growth media immediately prior to use.

For hemoglobin purification, 25 mL of fresh, heparinized human blood was centrifuged at 1500*g* at 4°C for 10 min to pellet the erythrocytes. Erythrocytes were washed three times in three pellet volumes of ice-cold sterile saline and subsequently lysed through resuspension in two pellet volumes of 50 mmol/L Tris pH 8.6, 2 mmol/L EDTA. Erythrocyte lysis was allowed to proceed for 30 min at room temperature, mixing periodically by gentle inversion. Cell debris was removed by centrifugation at 11000*g* for 30 min at 4°C. The supernatant was transferred to a fresh tube and solid NaCl was added (50 mg/mL), with mixing by inversion. The stroma was then precipitated by centrifugation at 11000*g* for 30 min at 4°C. The hemoglobin-containing supernatant was dialyzed overnight at 4°C against 50 mmol/L Tris pH 8.6, 1 mmol/L EDTA (Buffer A). The dialyzed hemoglobin was passed once through a 0.4 *μ*m syringe filter prior to purification via anion exchange on a Mono Q HR 16/10 column (GE Healthcare, Mississauga, ON, Canada). Dialyzed hemoglobin solution was loaded in 4–6 mL batches on the column and a gradient run using 50 mmol/L Tris pH 8.6, 1 mmol/L EDTA, 0.5 mol/L NaCl as Buffer B; hemoglobin fraction eluted between 50 and 100 mmol/L NaCl. The purified fractions were dialyzed into 50 mmol/L Tris pH 8.0 and sterilized by passage through a 0.4 *μ*m syringe filter. Purity was assessed using UV–Vis spectrometry. Briefly, a spectral scan was run between 200 and 800 nm, where a characteristic Soret peak at 415 nm, as well as distinct *α*_576 nm_ and *β*_541 nm_ bands, and a peak at 345 nm, were indicative of intact ferrous oxyhemoglobin. Hemoglobin concentration was determined using published extinction coefficients at 560 and 577 nm (Winterbourn et al. 1976) and was concentrated to 2 mmol/L using Amicon Ultra-15 centrifugation units (30 kDa NMWL; Millipore). Small aliquots were flash frozen in a dry ice–ethanol bath and stored at −80°C.

### Assessment of hemin and hemoglobin utilization by *S. lugdunensis*

In assessing hemin/hemoglobin utilization, single, isolated colonies of *S. lugdunensis* from overnight TSA plates were resuspended in 120 *μ*L RPMIC. 100 *μ*L of this suspension was used to inoculate 2 mL RPMIC with 0.1 *μ*mol/L EDDHA, which was grown for at least 4 h until OD_600_ was ∼1. Precultures were subcultured 1:400 into 2 mL aliquots of RPMIC with 1 *μ*mol/L EDDHA and either 1–500 nmol/L human hemoglobin, purified as described above, 20–1000 nmol/L hemin, prepared as described above, or 1 *μ*mol/L FeSO_4_. Cultures were incubated with shaking at 200 rpm, in 14-mL round-bottom polypropylene tubes. OD_600_ was assessed at 12, 24, and 36 h.

## Results

### Sequence analysis of key iron acquisition loci in *S. lugdunensis*

*S. aureus* contains several key iron acquisition loci that are well-characterized. These include the *isd* locus that promotes iron acquisition from heme and hemoglobin, the *sfa-hts* locus for synthesis and reentry of SA and the *sbn-sir* locus for synthesis and uptake of SB (Beasley and Heinrichs 2010; Hammer and Skaar 2011). The genome sequences of *S. lugdunensis* strains N920143 and HKU01-09 indicate that these strains possess *htsABC* genes downstream from an *sfa* locus (Fig. 1A), with predicted products that are highly similar to the *S. aureus* HtsABC proteins (Table 2). However, in contrast to *S. aureus*, the *S. lugdunensis sfa* locus in both strains contains a deletion of ∼3.3 kb that eliminates the *sfaA* and *sfaD* genes, as well as the promoter for the remaining *sfaBC* genes (Fig. 1A). This suggests that *S. lugdunensis* does not synthesize SA, yet may be able to utilize it as an iron source, via HtsABC, if SA were provided exogenously. In contrast to *S. aureus*,*S. lugdunensis* lacks the *sbn* operon, which encodes the products responsible for synthesizing SB. Interestingly, *S. lugdunensis* possesses homologs of the *S. aureus sirABC* genes (predicted products are highly similar to those from *S. aureus*, see Table 2), which encode a SB transporter, and these genes are situated immediately downstream of the *S. lugdunensis isd* locus (Fig. 1B). In comparison to *S. lugdunensis* N920143, strain HKU01-09 contains an exact, tandem duplication of a large region of DNA encompassing the *isd-sir* locus (Fig. 1B). Together, the sequence analysis of these loci suggests that *S. lugdunensis* should not be able to synthesize either SA or SB, in contrast to *S. aureus*, which synthesizes and secretes both staphyloferrin molecules. On the other hand, we would predict that *S. lugdunensis* should be able to transport iron via these siderophores, as well as acquire iron from heme/hemoglobin via Isd proteins.

**Table 2:** Similarity of iron-regulated proteins between *Staphylococcus aureus* and *Staphylococcus lugdunensis*.

Protein	Function	% ID/% TS
HtsA	Lipoprotein; receptor for ferric-staphyloferrin A (Fe-SA)	70/82
HtsB	Permease; specific for Fe-SA	60/74
HtsC	Permease; specific for Fe-SA	71/87
SfaB	Synthetase; completes SA synthesis by addition of citric acid to alpha amine of d-ornithine in citryl-d-ornithine intermediate	43/68
SfaC	Amino acid racemase; predicted to convert l-ornithine to d-ornithine	57/79
SirA	Lipoprotein; receptor for ferric-staphyloferrin B (Fe-SB)	85/92
SirB	Permease; specific for Fe-SB	84/94
SirC	Permease; specific for Fe-SB	74/84
IsdC	Wall anchored heme-binding protein	57/67
IsdE	Lipoprotein; receptor for heme	75/85
IsdB	Wall-anchored hemoglobin-binding protein	35/52
IsdA (Sa) vs. IsdJ (Sl)	Wall-anchored heme-binding protein	19/29
IsdA (Sa) vs. IsdK (Sl)	Wall-anchored heme-binding protein	14/31

ID, identity; TS, total similarity; Sa, *S. aureus*; Sl, *S. lugdunensis*.

### *S. lugdunensis* grows poorly in iron-restricted media, owing to a lack of siderophore production

**Figure 2:**
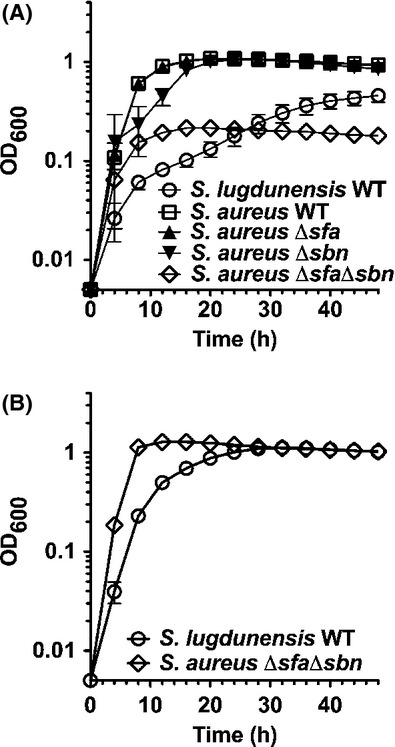
*Staphylococcus lugdunensis* grows poorly in iron-restricted growth media. (A) Growth kinetics comparing *S. lugdunensis* to that of *Staphylococcus aureus*WT and staphyloferrin A (*sfa*) and staphyloferrin B (*sbn*)-deficient mutants in C-TMS with 20% serum. (B) The growth deficiencies of *S. lugdunensis* and the *S. aureus* staphyloferrin-deficient mutant in iron-restricted media are complemented with addition of 100 *μ*mol/L FeCl_3_. All data points represent average values for at least three independent biological replicates, and error bars represent standard deviation from the mean.

Our previous studies showed that in comparison to wild-type *S. aureus*, mutants lacking the ability to synthesize the two staphyloferrin siderophores (i.e., *sfa sbn*) grow poorly in iron-restricted media containing transferrin or serum (as a source of transferrin) (Beasley et al. 2009, 2011b). Given that *S. lugdunensis* genomic information suggests an inability to produce a staphyloferrin siderophore, we compared the growth of *S. lugdunensis* to that of *S. aureus* and its staphyloferrin-deficient mutants in iron-restricted media. In comparison to wild-type *S. aureus*,*S. lugdunensis* grew poorly in C-TMS with 20% serum (Fig. 2A) or transferrin (data not shown) and, even after extended incubation periods, never reached a final biomass equivalent to that of *S. aureus*. Indeed, in these culture conditions, *S. lugdunensis* HKU01-09 (strain HKU01-09 was used throughout this study) grew at a slower rate than the *S. aureus sfa sbn* mutant. We demonstrated that the growth deficiency of *S. lugdunensis* and *S. aureus sfa sbn* was due to a deficiency in the ability to scavenge trace amounts of iron, as supplementation of the growth medium with 100 *μ*mol/L FeCl_3_ promoted rapid growth of both (Fig. 2B).

**Figure 3:**
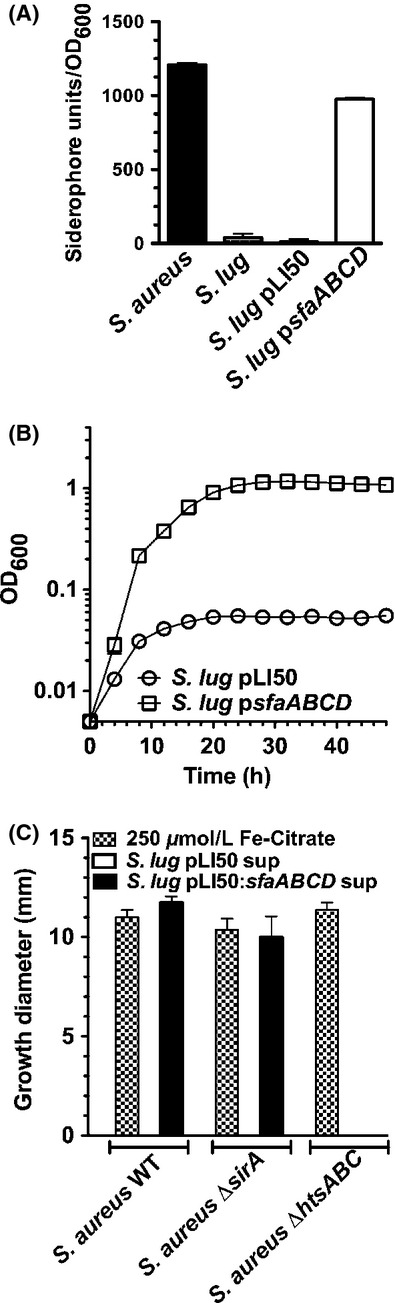
Introduction of a complete *sfa* locus from *Staphylococcus aureus* into *Staphylococcus lugdunensis* leads to SA production and growth in iron-restricted media. (A) Chrome azurol S (CAS) assay demonstrates that culture supernatants of *S. lugdunensis* lack siderophore activity, whereas introduction of the plasmid carrying the *S. aureus sfaA-D* genes into *S. lugdunensis* allows for the production and secretion of siderophore into culture supernatants. (B) Growth of *S. lugdunensis*WT strain containing either vehicle (pLI50) or plasmid carrying the staphyloferrin A biosynthetic genes (*sfaA-D*) in C-TMS plus 20% serum. (C) Agar plate bioassays demonstrating that the culture supernatant of *S. lugdunensis* does not promote the growth of iron-restricted *S. aureus* strains, while the supernatant of *S. lugdunensis* carrying the *sfa* gene cluster, which generates SA, can promote the growth of iron-restricted *S. aureus* in an *hts* (encodes the SA transporter)-dependent manner. Ferric citrate was used as a positive control in the experiment. All data points represent average values for at least three independent biological replicates, and error bars represent standard deviation from the mean.

In further support of the bioinformatics analyses indicating that *S. lugdunensis* is incapable of staphyloferrin production, the culture supernatants of *S. lugdunensis*, grown in C-TMS, tested negative in the chrome azurol S assay (Fig. 3A), indicating a lack of secreted iron-binding molecules. This is in contrast to the positive result obtained for *S. aureus* culture supernatants grown in the same medium and under the same conditions.

Cotton et al. (2009) have previously demonstrated that the cloned *sfa* gene cluster allowed heterologous synthesis and secretion of SA in *E. coli*. Knowing this, we used our plasmid containing the *sfaA-D* genes, which was previously used to complement the *sfa* deletion in *S. aureus* (Beasley et al. 2009), and introduced it into *S. lugdunensis*. The chrome azurol S assay was used to demonstrate that when we grew the recombinant *S. lugdunensis* strain in iron-restricted media, we could detect robust siderophore activity compared to wild-type or the strain carrying the vector control (Fig. 3A).

Despite not synthesizing SA, *S. lugdunensis* has *htsABC* homologs that, in *S. aureus*, are required for utilization of ferric-SA complexes. To test the possibility that *S. lugdunensis* can utilize SA, we demonstrated that the *sfa*-complemented strain displayed enhanced growth in C-TMS media containing 20% serum, as compared to wild-type *S. lugdunensis* carrying the empty control plasmid (Fig. 3B). In support of the result that we could not detect iron-binding molecules in the iron-restricted culture supernatant of *S. lugdunensis*, we demonstrated that concentrated culture supernatants of *S. lugdunensis* were incapable of enhancing iron-restricted growth of *S. aureus* (Fig. 3C). Importantly, concentrated culture supernatant from *S. lugdunensis* containing the *S. aureus sfa* gene cluster could readily promote growth of wild-type and *sirA*-deficient *S. aureus*, but not an *htsABC*-mutant *S. aureus* strain. This is in agreement with the notion that *S. lugdunensis* expressing the *S. aureus sfa* genes was making SA and using it to promote enhanced growth in iron-restricted media.

### *S. lugdunensis HtsABC* and *SirABC* function as transporters for SA and SB, respectively

**Figure 4:**
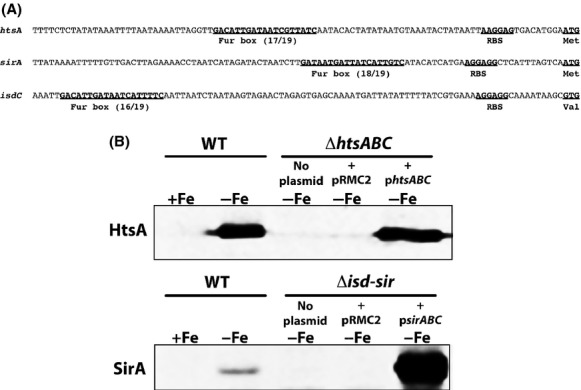
Expression of *Staphylococcus lugdunensis* HtsA and SirA homologues is iron-regulated. (A) Identification of Fur-boxes upstream of the *htsA*,*sirA* and *isdC* genes in *S. lugdunensis*. Numbers represent the number of identical bases between the 19-bp Fur boxes of *S. aureus* and *S. lugdunensis*. (B) Western blots demonstrating iron-regulated expression of HtsA and SirA, and confirmation of mutations and complementation, where pRMC2 is the vehicle control. Cultures were grown in C-TMS with (+Fe) or without (−Fe) addition of FeCl_3_ (25 *μ*mol/L). Mutant samples were all grown in C-TMS without addition of iron.

Promoters containing putative Fur box sequences are found upstream of the *htsABC* and *sirABC* operons in *S. lugdunensis* (Fig. 4A), suggesting that each operon is iron-regulated in a Fur-dependent fashion. Due to high amino acid sequence similarity of the *S. lugdunensis* HtsA and SirA with the *S. aureus* homologs (see Table 2), antibodies raised against *S. aureus* HtsA and SirA were used to successfully demonstrate iron-regulated expression of the *S. lugdunensis* proteins (Fig. 4B). We next deleted the *htsABC* genes from the *S. lugdunensis* genome and complemented the genes *in trans* by cloning the *htsABC* genes from *S. lugdunensis* back into the mutant strain. As shown in Fig. 4B, the Δ*htsABC* strain failed to express HtsA while the complemented strain showed restored expression of HtsA.

To generate a *S. lugdunensis* mutant lacking both copies of *sirABC*, we deleted the entire tandemly duplicated region (Fig. 1B) from the genome, a deletion of ∼65 kb; we refer to this mutant strain as Δ*isd-sir*. As expected, the mutant failed to express SirA (Fig. 4B). Complementation of the Δ*isd-sir* strain with the *sirABC* genes from *S. lugdunensis* restored expression of SirA (Fig. 4B).

**Figure 5:**
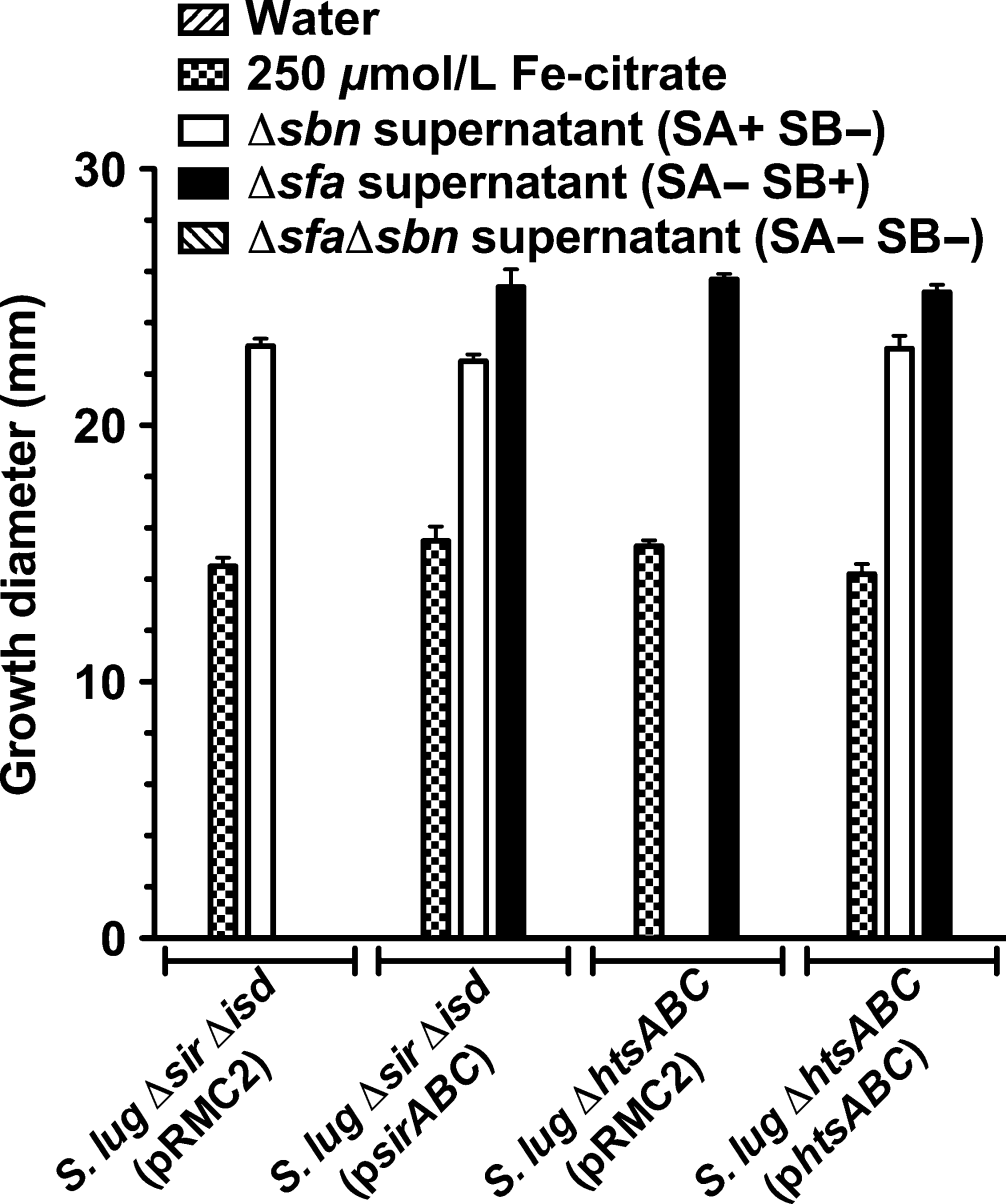
Plate bioassays demonstrate that *Staphylococcus lugdunensis* HtsABC and SirABC are required for uptake of staphyloferrin A and staphyloferrin B, respectively. Water and ferric citrate were used as negative and positive controls, respectively. Supernatant extracts supplied were those of *Staphylococcus aureus* mutants that secrete SA (*sbn* mutant), SB (*sfa* mutant) or neither SA or SB (*sfa sbn* mutant). All data points represent average values for at least three independent biological replicates, and error bars represent standard deviation from the mean.

With mutant and complemented strains in hand, we used them to test the ability of the strains to utilize the two staphyloferrin siderophores produced by *S. aureus*. As shown in Fig. 5, the ability of *S. lugdunensis* strains to utilize SA and SB, whether provided in concentrated culture supernatants from *S. aureus* strains, or as enzymatically synthesized and HPLC-purified molecules (data not shown), was absolutely dependent on the expression of *htsABC* and *sirABC*, respectively. Moreover, while the growth of *S. lugdunensis* was enhanced when the intact *S. aureus sfa* gene locus was introduced on a plasmid (Fig. 3B), the same plasmid was incapable of complementing the iron-restricted growth defect of the *S. lugdunensis* Δ*htsABC* mutant (data not shown). Together, these data prove that both the HtsABC and the SirABC transporters are functional in *S. lugdunensis*.

### *S. aureus* enhances *S. lugdunensis* growth in a staphyloferrin-dependent manner

**Figure 6:**
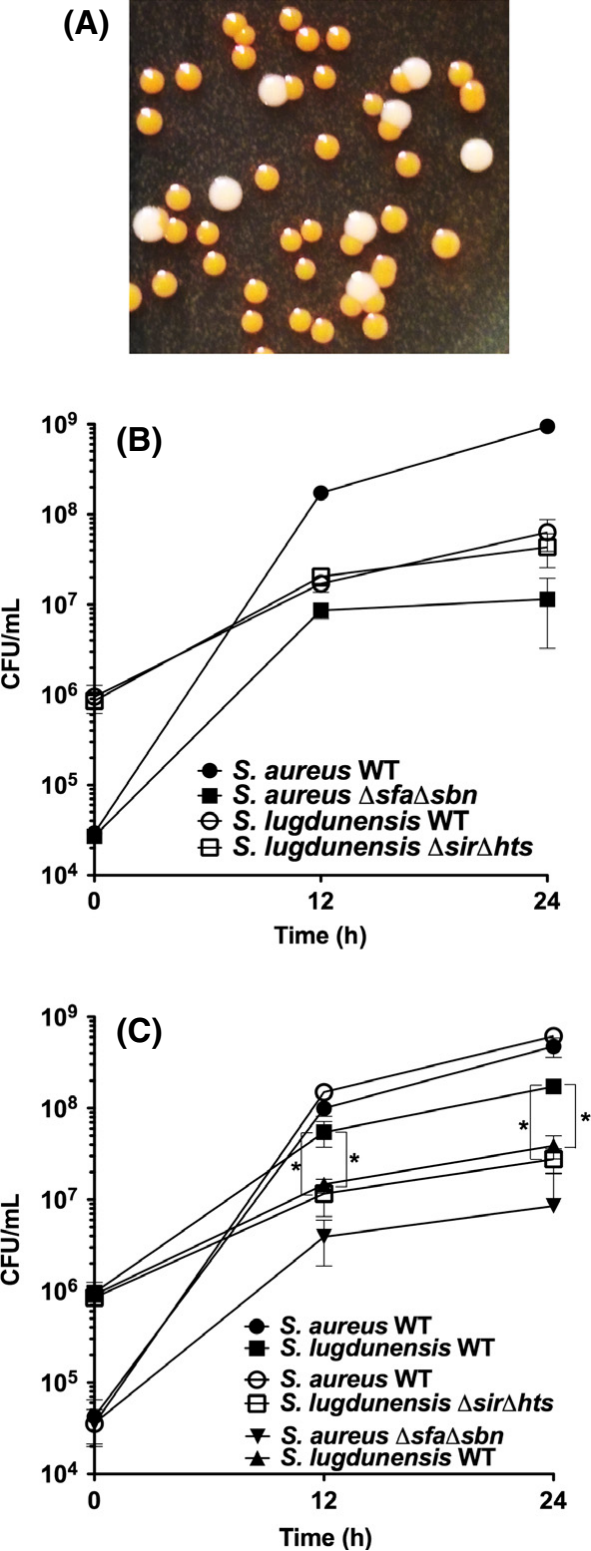
Coculture experiments demonstrate that *Staphylococcus aureus*-produced siderophores can enhance the iron-restricted growth of *Staphylococcus lugdunensis*. (A) Picture of colonies of *S. aureus* RN6390 (large and white) and *S. lugdunensis* (smaller and yellow) growing on a TSB plate after 24 h of incubation at 37°C, followed by 48 h of incubation at room temperature. (B) Growth of individual strains in C-TMS + 20% serum was monitored for CFU/mL at 12 and 24 h timepoints. (C) Growth of strains in cocultures with the pairs of strains grouped as indicated. All data points represent average values for at least three independent biological replicates, and error bars represent standard deviation from the mean. The Student's unpaired *t*-test was used to define statistical significance for the CFU values between strains as indicated by the brackets. *P < 0.0001.

As shown above, we demonstrated that exogenously added staphyloferrins could promote the growth of *S. lugdunensis*. Knowing this, we next decided to test whether *S. aureus*, which secretes both SA and SB, could augment the growth of *S. lugdunensis* if they were cultured together in iron-restricted growth media. We first optimized culture conditions so as to be able to easily discern colonies of *S. aureus* RN6390 from those of *S. lugdunensis* HKU09-01 on TSA (Fig. 6A) (see Experimental Procedures for details).

Figure 6B demonstrates that, when cultured in C-TMS containing 20% serum, wild-type *S. aureus* consistently grows from 2 × 10^4^ CFU/mL to ∼1 × 10^9 ^CFU/mL within 24 h, whereas the isogenic staphyloferrin-deficient mutant grows to a density of only 1 × 10^7^ CFU/mL over the same time frame. *S. lugdunensis*, on the other hand, inoculated at a higher cell density of 1 × 10^6^ CFU/mL, only reaches a final cell density of less than 1 × 10^8^ CFU/mL in 24 h. The isogenic *S. lugdunensis isd-sir hts* mutant displays identical growth kinetics in these culture conditions.

For coculture experiments, in pilot studies, we found that we needed to inoculate *S. lugdunensis* at much higher cell densities than *S. aureus* because *S. aureus* grows significantly faster than *S. lugdunensis* in these culture conditions. Data displayed in Figure 6C demonstrate that when wild-type *S. aureus* is cocultured with wild-type *S. lugdunensis*,*S. lugdunensis* grew to much higher density, ∼2 × 10^8^, than when cultured on its own (c.f. Fig. 6B vs. Fig. 6C). We next demonstrated that this growth enhancement was due to the use of the *S. aureus*-produced staphyloferrins by *S. lugdunensis* by use of two complementary experiments (Fig. 6C). First, wild-type *S. aureus* did not enhance the growth of the *S. lugdunensis* Δ*isd-sir* Δ*htsABC* mutant like it did wild-type *S. lugdunensis*. Second, staphyloferrin-deficient *S. aureus* had no effect on the growth of wild-type *S. lugdunensis*. Together, these data provide convincing evidence that *S. aureus*, through production of SA and SB, enhances the iron-restricted growth of *S. lugdunensis* in a HtsABC-and SirABC-dependent manner, respectively.

### The *S. lugdunensis isd-sir* mutant is attenuated for utilization of heme and hemoglobin

**Figure 7:**
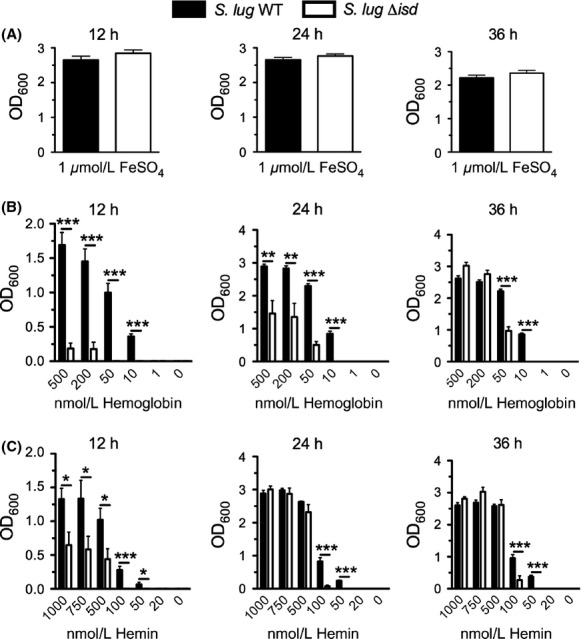
Growth of *Staphylococcus lugdunensis*WT and its isogenic Δ*isd* mutant using iron, hemoglobin or heme as a sole iron source. Growth of the Δ*isd-sir* mutant was compared to that of WT strain HKU09-01 at 12-, 24-and 36-h timepoints in RPMIC containing FeSO_4_ (A) varying concentrations of human hemoglobin, (B) or varying concentrations of bovine hemin, (C) as the sole iron source. All data points represent average values for at least three independent biological replicates, and error bars represent standard deviation from the mean. Statistical significance was determined using the Student's unpaired *t*-test; *P < 0.05; **P < 0.01; ***P < 0.0001.

Having constructed a complete *isd* locus deletion strain of *S. lugdunensis* HKU01-09, we were therefore positioned to test the mutant for its ability to utilize heme and hemoglobin as sole sources of iron. Zapotoczna et al. (2012) have also recently generated *isdB* and *isd* locus deletion mutants in *S. lugdunensis* strain N920143 and shown that the mutants were impaired for growth on hemoglobin compared to wild type. These experiments were performed using a single concentration of hemoglobin, and heme as a sole iron source was not tested. We took a more comprehensive approach by examining the growth of the Δ*isd-sir* deletion mutant in iron-starved media containing a range of heme and hemoglobin concentrations, in media that contained enough of the nonmetabolizable iron chelator EDDHA to completely restrict growth unless an iron source was added. As shown in Figure 7A, the Δ*isd-sir* mutant is not debilitated for use of FeSO_4_ as an iron source. In Figure 7B, it is notable that the Δ*isd-sir* mutant is attenuated for growth at all concentrations of hemoglobin tested (500 nmol/L down to 10 nmol/L; 1 nmol/L hemoglobin is insufficient to promote growth of wild type under these conditions), especially at 12 h but also by the 24 h timepoint. By 36 h, it was apparent that hemoglobin was beginning to promote the growth of the mutant, especially at the higher hemoglobin concentrations. Notably, at the low nM concentrations of hemoglobin (i.e., below 50 nmol/L), the Δ*isd-sir* mutant is significantly attenuated for growth, compared to wild type, even through 36 h of incubation.

A similar growth pattern was observed for the Δ*isd-sir* mutant when hemin was used as the sole iron source (Fig. 7C). It is apparent that the *isd-sir* locus provides a significant growth advantage to *S. lugdunensis* at early stages of growth at all concentrations tested (up to 1 *μ*mol/L) and continues to provide a significant growth advantage to the cells through 36 h of incubation in the presence of hemin at concentrations below 100 nM (Fig. 7C).

## Discussion

*S. lugdunensis* is a relatively recently recognized bacterium that is both a commensal, and an important human pathogen, capable of causing serious infections such as aggressive native valve infective endocarditis (IE) (Frank et al. 2008). That IE caused by *S. lugdunensis* can occur independent of indwelling medical devices differentiates *S. lugdunensis* from other CoNS, and makes it worthy of investigation. Molecular studies of *S. lugdunensis* are in their infancy and, thus, there is a paucity of information concerning important virulence factors that underpin the potential of this opportunistic pathogen to cause severe and invasive infections. The molecular mechanisms of iron acquisition that are key to the biology and infectivity of this bacterium are essentially unknown. Zapotoczna et al. (2012) examined the role of the iron-regulated surface determinant system in the utilization of hemoglobin and found that, in strain N920143, the Isd-dependent heme/hemoglobin utilization system is functional by demonstrating that an *isd* deletion mutant and an *isdB* mutant were both slightly debilitated for growth on hemoglobin as a sole iron source. Moreover, *S. lugdunensis* IsdG, like its *S. aureus* counterpart, degrades heme to staphylobilin and free iron (Haley et al. 2011). In this study, we furthered these findings by demonstrating that the tandemly duplicated *S. lugdunensis isd* locus in strain HKU09-01 was required for promoting early and rapid growth on a wide range of hemoglobin and heme concentrations ranging from 10 to 500 nmol/L and 50–1000 nmol/L, respectively (Fig. 7B and C), eventually leading to increased bacterial biomass that was sustained through 36 h of incubation, especially at the lower concentrations. It is worth noting that these low concentrations of hemoglobin and heme are physiologically relevant, as these molecules are removed from circulation in the liver subsequent to being quickly bound by haptoglobin and hemopexin, respectively.

*S. lugdunensis* is severely attenuated for growth in iron-restricted media that do not contain a readily utilizable iron source such as heme or hemoglogin. In media containing serum or transferrin, for example, *S. lugdunensis* is severely compromised for growth compared to *S. aureus*. We have shown that this growth defect is readily corrected with the introduction of a functional *S. aureus* SA biosynthetic locus into *S. lugdunensis*, where the recombinant strain synthesizes and secretes readily detectable amounts of SA into the culture supernatant. Based upon previous studies, it is known that the *sfa* gene cluster from *S. aureus* is both necessary, based on the phenotype of *sfa* mutants (Beasley et al. 2009) and sufficient, based on heterologous cloning in *E. coli* (Cotton et al. 2009) for SA synthesis.

That the *sfa* deletion is found in both *S. lugdunensis* strains where genome sequence is available suggests that this deletion may be common to the species. The deletion (see Fig. 1) removes the promoter for each of the two transcripts *sfaD* and *sfaABC*, and also completely removes the *sfaA* and *sfaD* genes, encoding the putative SA efflux pump (SfaA) and one of the synthetases that joins a citrate molecule to the *δ*-amine of d-ornithine to form the first amide intermediate of SA (Cotton et al. 2009). Studies in the early 1990s identified SA in culture supernatants of many species of staphylococci, and that SB was found in supernatants of fewer species (Meiwes et al. 1990; Haag et al.^1994^). Of the species tested, which did not include a *S. lugdunensis* representative, only *S. sciuri* and *S. hominis* were found incapable of producing either siderophore, but only one strain was tested from each of these species and the result may not reflect the capabilities of the species overall. Based on available genomic data, on the other hand, *S. lugdunensis* (genome data exist for strains N920143 and HKU09-01) appears to be unique amongst the staphylococci in its inability to synthesize at least one of the two staphyloferrin molecules. This would imply that if other *S. lugdunensis* strains also lack these loci, the species causes opportunistic infections independent of siderophore production, and would presumably rely on either heme acquisition or uptake of xenosiderophores to satisfy its iron requirements throughout the various stages of colonization and infection. Genomic information identifies that *S. lugdunensis* has homologs of *fhuCBG* and *sstABCD* where predicted products share high levels of similarity with those in *S. aureus*. This would suggest that *S. lugdunensis*, like we have previously reported for *S. aureus* (Sebulsky et al. 2000; Beasley et al. 2011b), is capable of using hydroxamates (via FhuCBG) and catechols/catecholamines (via SstABCD) as a means to acquire iron. The *S. lugdunensis* FhuC ATPase shares greater than 95% similarity with its *S. aureus* counterpart and we hypothesize that this ATPase is, like in *S. aureus* (Speziali et al. 2006), important for providing the energy for not only the uptake of hydroxamate siderophores, but also the staphyloferrins through HtsABC and SirABC.

Interestingly, despite its noted ability to cause serious infection in humans, *S. lugdunensis* N920143 was reported to cause only very mild infection in a rat model of endocarditis, much milder than that which would be caused by equivalent CFUs of *S. aureus* (Heilbronner et al. 2013). In pilot experiments, we, too, noted a relative lack of virulence of *S. lugdunensis* HKU09-01, using the mouse model of hematogenous spread. Mice challenged with up to 1 × 10^8^ CFUs showed no overt signs of illness, and continued to gain weight, despite detectable counts in the kidneys for at least 7 days following bacterial challenge via tail vein. This contrasts from the course of disease that would be caused by a much lower dose of *S. aureus*. As noted previously (Frank et al. 2008; Tse et al. 2010; Heilbronner et al. 2011, 2013), the *S. lugdunensis* genome indicates an absence of orthologs of well-centeracterized *S. aureus* toxin and immune evasion encoding genes, suggestive of a limited capacity to cause severe disease, at least in comparison to *S. aureus*.

That *S. lugdunensis* has retained the transport machinery for both SA and SB is interesting, despite an inability to synthesize either siderophore. This may represent a mechanism for scavenging these iron-binding molecules produced by other species of staphylococci, including *S. aureus*; recall from above that many species of staphylococci produce at least SA. Our data show that coculture of *S. aureus* with *S. lugdunensis* in the same iron-limited growth media enhanced the growth of *S. lugdunensis* by at least one log. This growth enhancement was due to *S. lugdunensis*’ ability to scavenge the *S. aureus* staphyloferrins in an Hts-and Sir-dependent manner. We speculate that this is a viable ‘opportunistic’ strategy used by *S. lugdunensis* in vivo, and the growth-promoting species could be any one of a number of the species of staphylococci that produce at least one of the staphyloferrin molecules. Although *S. lugdunensis* predominantly inhabits lower parts of the human body (i.e., the perineum) (Böcher et al. 2009) and *S. aureus* largely inhabits the nares, both species are present to some degree over the entire external surface of the body (Bellamy and Barkham 2002; Bieber and Kahlmeter 2010; Edwards et al. 2012). Indeed, *S. lugdunensis* is recovered with other bacteria in ∼60% of infections, including co-occurrence with *S. aureus* and other staphylococci (Herchline and Ayers 1991). It may be that over time *S. lugdunensis* has evolved to simply steal siderophores produced by other species of bacteria, including *S. aureus*, and in these situations is more capable of causing opportunistic infections of humans.
